# Detection of Exosomal miRNAs in the Plasma of Melanoma Patients

**DOI:** 10.3390/jcm4121957

**Published:** 2015-12-17

**Authors:** Susan R. Pfeffer, Kenneth F. Grossmann, Pamela B. Cassidy, Chuan He Yang, Meiyun Fan, Levy Kopelovich, Sancy A. Leachman, Lawrence M. Pfeffer

**Affiliations:** 1 Department of Pathology and Laboratory Medicine, Center for Cancer Research, University of Tennessee Health Science Center, 19 South Manassas Street, Memphis, TN 38163, USA; spfeffer@uthsc.edu (S.R.P.); cyang@uthsc.edu (C.H.Y.); mfan2@uthsc.edu (M.F.); 2 Department of Oncology, University of Utah, Salt Lake City, UT 84112, USA; kenneth.grossmann@hci.utah.edu; 3 Department of Dermatology, Oregon Health & Science University, Portland, OR 97239, USA; cassidyp@ohsu.edu (P.B.C.); leachmas@ohsu.edu (S.A.L.); 4 Department of Medicine, Weill Cornell College of Medicine, New York, NY 10065, USA; kopelovichl@gmail.com

**Keywords:** miRNAs, melanoma, exosomes, metastasis

## Abstract

MicroRNAs (miRNAs) are a class of 22–25 nucleotide RNAs that control gene expression at the post-transcriptional level. MiRNAs have potential as cancer biomarkers. Melanoma is a highly aggressive form of skin cancer accounting for almost 4% of cancers among men and women, and ~80% of skin cancer-related deaths in the US. In the present study we analyzed plasma-derived exosomal miRNAs from clinically affected and unaffected familial melanoma patients (CDKN2A/p16 gene carriers) and compared them with affected (nonfamilial melanoma) and unaffected control subjects in order to identify novel risk biomarkers for melanoma. Intact miRNAs can be isolated from the circulation because of their presence in exosomes. A number of differentially regulated miRNAs identified by NanoString human V2 miRNA array were validated by quantitative PCR. Significantly, miR-17, miR-19a, miR-21, miR-126, and miR-149 were expressed at higher levels in patients with metastatic sporadic melanoma as compared with familial melanoma patients or unaffected control subjects. Surprisingly, no substantial differences in miRNA expression were detected between familial melanoma patients (all inclusive) and unaffected control subjects. The miRNAs differentially expressed in the different patient cohorts, especially in patients with metastatic melanoma, may play important roles in tumor progression and metastasis, and may be used as predictive biomarkers to monitor remission as well as relapse following therapeutic intervention.

## 1. Introduction

MicroRNAs (miRNAs) are an abundant class of small RNAs that control gene expression at the post-transcriptional level through degradation or repression of mRNA translation [1]. MiRNAs are able to regulate the expression of multiple targets by binding to the 3′-untranslated regions of genes. Emerging evidence suggests that miRNAs are involved in critical biological processes, including development, differentiation, apoptosis, proliferation and antiviral defense [2]. Most importantly, aberrant expression of miRNAs appears to be causatively linked to the pathogenesis of cancer [3]. Thus, miRNAs have potential as risk biomarkers, particularly following therapeutic intervention.

Exosomes are small (30–100 nm) extracellular vesicles that are produced by a wide variety of cell types, including tumor cells [4]. Although exosomes were originally considered to be cellular waste products, recent studies have demonstrated that they promote intercellular communication and immunoregulatory processes by shuttling proteins, lipids, and miRNAs between cells [5,6]. Moreover, intact miRNAs can be isolated from the circulation in significant quantities despite the presence of high levels of RNase activity because of their presence in exosomes [7,8]. The remarkable stability of circulating exosomal miRNAs makes them candidates to monitor disease progression in a variety of cancers.

Skin cancer is the most common human cancer. The incidence of melanoma, the most lethal skin cancer, is one of the few cancers in the U.S. that continues to rise [9]. Melanoma is a highly aggressive form of skin cancer that accounts for almost 5% of cancers among men and women, and ~80% of skin cancer-related deaths in the US. The clustering of several melanomas within a single family, several independent primary melanomas in a single individual, and co-incidence of several melanomas and other cancers such as pancreatic cancer in the same family are all associated with inheritance of germline mutations in a high-penetrance melanoma susceptibility gene [10]. The most common high-penetrance melanoma predisposition gene is cyclin-dependent kinase inhibitor 2A, which encodes two independent predisposition genes, CDKN2A/p16 and CDKN2A/ARF. CDKN2A mutations occur in approximately 20%–40% of melanoma-prone families worldwide [11,12]. Variable rates of mutation have been found in sporadic melanomas, in some studies being as high as 50% in primary lesions [13]. The CDKN2A gene locus generates two proteins through alternate reading frames: p16^INK4a^ and p14^arf^. The p16^INK4a^ protein binds to CDK4 and CDK6, inhibiting their ability to phosphorylate the retinoblastoma protein. The p14^arf^ protein stabilizes the tumor suppressor protein p53. Collectively, these CDKN2A gene products are potent tumor suppressors that play distinct but critical roles in cell cycle progression and apoptosis [14]. Heterozygous loss of p16^INK4a^ function is sufficient to confer a 67% lifetime risk of melanoma [15].

According to the National Cancer Institute, in 2015 an estimated 76,100 new melanoma cases will be diagnosed and 9710 deaths will occur in the US. Identifying early biomarkers for melanoma would enable discovery of potential targets and presumably agents for early intervention in persons at risk of developing melanoma. A noninvasive screening tool to identify patients with a predisposition to melanoma is presently lacking. Whole blood holds several advantages as a biomarker specimen, most notably because sampling and processing is much simpler than that of skin. In this regard it holds significant potential as a point-of-care test, which would be attractive in determining an ideal cancer-screening tool.

We previously showed that gene expression profiles are altered in phenotypically normal skin fibroblasts from familial melanoma families with distinct CDKN2A/p16 mutations (DKN2A:c.377T>A (p.V126D) and CDKN2A:c.259G>T (p.R87P)) when compared to skin fibroblasts from normal controls [16]. Furthermore, UV-irradiation of skin fibroblasts from such familial melanoma cohorts resulted in specific alterations in the expression of genes that regulate cell cycle and DNA damage response, and similar alterations in gene expression were also observed in melanoma lesions. In the present study, we investigated whether exosomal-derived miRNAs in the plasma from both clinically symptomatic and asymptomatic familial (CDKN2A:c.377T>A (p.V126D)) and sporadic melanoma patients, including unaffected family members, may be used as prognostic biomarkers to identify individuals at high risk of developing melanoma. However, this proof of principle experiment did not identify miRNAs specifically dysregulated in plasma-derived exosomes from familial melanoma patients. Nonetheless, several miRNAs were differentially expressed in patients with metastatic disease, not only in melanoma tumor tissue but also in plasma-derived exosomes. This result substantiates the finding of miRNA dysregulation in metastatic melanoma [17,18,19]. These findings form the basis for future studies on their applicability as diagnostic and prognostic biomarkers in melanoma.

## 2. Results

### 2.1. Characterization of miRNA Expression in Plasma-Derived Exosomes from Patients with a Predisposition to Melanoma and Patients with Metastatic Melanoma

We performed miRNA profiling on RNA prepared from plasma-derived exosomes from four specific patient cohorts. The general patient information is shown in Table 1. Cohort A comprised 8 clinically affected individuals from a single large family, who carried a CDKN2A/p16 (CDKN2A:c.377T>A (p.V126D)) mutation but were free of disease at the time of blood draw. Cohort B comprised 5 individuals from the same family as in Cohort A with CDKN2A/p16 mutations, but with no history of melanoma at the time of this study. Cohort C comprised 13 spouse controls in the same kindred as A and B above, and Cohort D consisted of 10 non-related metastatic melanoma patients with currently active disease. We hypothesized that genetically predisposed individuals such as those who carried a CDKN2A/p16 mutation might share the expression profile with individuals having sporadic metastatic melanoma.

**Table 1:** General patient information.

Cohort	Age	Gender	p16 Mutation Status	Melanoma Diagnosis
A1	37	M	377T>A (p.V126D)	N
A2	40	M	377T>A (p.V126D)	N
A3	37	M	377T>A (p.V126D))	N
A4	46	M	377T>A (p.V126D)	N
A5	64	F	377T>A (p.V126D)	N
B1	86	M	377T>A (p.V126D)	Y
B2	56	M	377T>A (p.V126D)	Y
B3	83	M	377T>A (p.V126D)	Y
B4	66	F	377T>A (p.V126D)	Y
B5	50	M	377T>A (p.V126D)	Y
B6	39	F	377T>A (p.V126D)	Y
B7	42	F	377T>A (p.V126D)	Y
B8	52	F	377T>A (p.V126D)	Y
C1	68	M	negative	N
C2	40	F	negative	N
C3	41	M	negative	N
C4	46	M	negative	N
C5	45	F	negative	N
C6	36	M	negative	N
C7	58	M	negative	N
C8	63	M	negative	N
C9	88	M	negative	N
C10	34	F	negative	N
C11	36	F	negative	N
C12	40	F	negative	N
C13	68	F	negative	N
D1	50	M	Not tested	met mel
D2	48	F	Not tested	met mel
D3	38	M	Not tested	met mel
D4	55	M	Not tested	met mel
D5	34	M	Not tested	met mel
D6	81	M	Not tested	met mel
D7	82	F	Not tested	met mel
D8	40	M	Not tested	met mel
D9	40	M	Not tested	met mel
D10	55	M	Not tested	met mel

**Figure 1:**
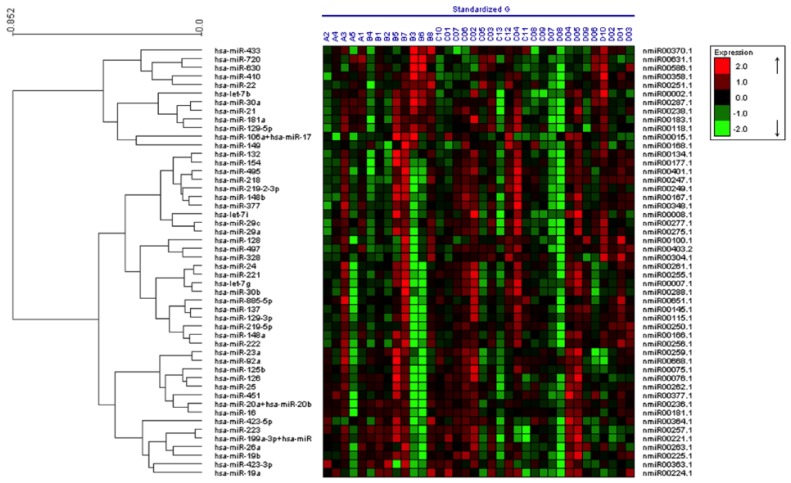
Characterization of miRNA expression in plasma-derived exosomes from individuals with a genetic predisposition to melanoma (all inclusive), spouse controls and patients with sporadic metastatic melanoma. RNA was prepared from plasma-derived exosomes from the patient cohorts listed in Table 1, and miRNA expression profiling was conducted on the nCounter Analysis System using the human V1 miRNA assay kit.

In brief, the miRNA expression data from the 36 samples was analyzed with nSolver software to identify alterations in miRNA expression. Among the ~700 human miRNAs examined, 75 miRNAs were detected in plasma-derived exosomes from more than half of the patients. The 50 miRNAs that showed highest total reads (most abundant) in the exosomes of the 36 patient samples were then subjected to unsupervised hierarchal clustering with the expression heat maps of the individual patient samples shown in Figure 1. The twenty most variable miRNAs among all samples were then further validated by qPCR analysis to examine their differential expression within the four patient cohorts described in Table 1. These miRNAs included let-7b, let-7g, miR-17, miR-19a, miR-19b, miR-20b, miR-21, miR-23a, miR-29a, miR-92a, miR-125b, miR-126, miR-128, miR-137, miR-148a, miR-149, miR-199a, miR-221, miR-222 and miR-423 (Table 2).

**Table 2:** Primers used for miRNA expression.

hsa-let-7b	TGAGGTAGTAGGTTGTGTGGTT
hsa-let-7g-5p	TGAGGTAGTAGTTTGTACAGTT
hsa-miR-125b	TCCCTGAGACCCTAACTTGTGA
hsa-miR-126	TCGTACCGTGAGTAATAATGCG
hsa-miR-128	TCACAGTGAACCGGTCTCTTT
hsa-miR-137	TTATTGCTTAAGAATACGCGTAG
hsa-miR-148a	AAAGTTCTGAGACACTCCGACT
hsa-miR-149	TCTGGCTCCGTGTCTTCACTCCC
hsa-miR-17	CAAAGTGCTTACAGTGCAGGTAG
hsa-miR-199a-5p	CCCAGTGTTCAGACTACCTGTTC
hsa-miR-19a	TGTGCAAATCTATGCAAAACTGA
hsa-miR-19b	TGTGCAAATCCATGCAAAACTGA
hsa-miR-20b	TAAAGTGCTTATAGTGCAGGTAG
hsa-miR-21	TAGCTTATCAGACTGATGTTGA
hsa-miR-221	AGCTACATTGTCTGCTGGGTTTC
hsa-miR-222	AGCTACATCTGGCTACTGGGT
hsa-miR-23a	ATCACATTGCCAGGGATTTCC
hsa-miR-29a	TAGCACCATCTGAAATCGGTTA
hsa-miR-423-5p	TGAGGGGCAGAGAGCGAGACTTT
hsa-miR-92a	TATTGCACTTGTCCCGGCCTGT

### 2.2. Detection of Circulating miRNA in Plasma-Derived Exosomes

Since there are no known control or house-keeping microRNAs in exosomes, we adopted the strategy of using spiked-in *C. elegans* miRNAs directly into Qiazol prior to RNA extraction as normalizing controls [20]. To determine whether our miRNA assays by qPCR were within the linear range of detection, a reference standard Cel-39 was spiked into Qiazol at 0.05 fmol/mL and 0.0005 fmol/mL prior to RNA extraction, and the expression of miR-21, miR-92b and miR-126 in plasma-derived exosomes was determined. These assays demonstrated the appropriate miRNA expression levels relative to the known quantity of spiked-in Cel39, demonstrating that our qPCR assay conditions for miRNAs were within the linear range.

**Figure 2:**
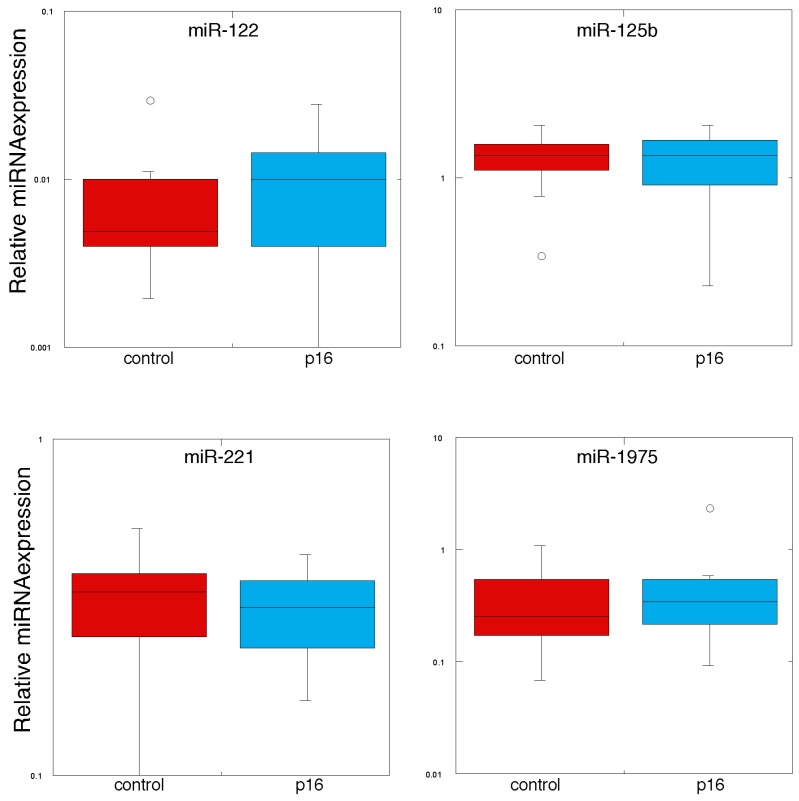
MiRNA expression in plasma-derived exosomes from individuals with the p16 mutation and normal volunteers. RNA was prepared from plasma-derived exosomes from individuals with the p16 mutation (CDKN2A:c.377T>A (p.V126D)) and normal volunteers. MiRNA expression was determined by qPCR (*n* = 3) and normalized to the spiked-in levels of Cel39.

**Figure 3:**
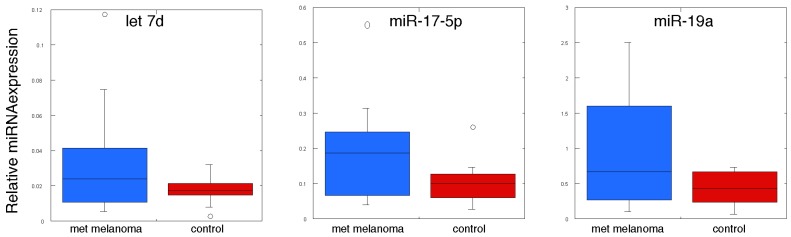

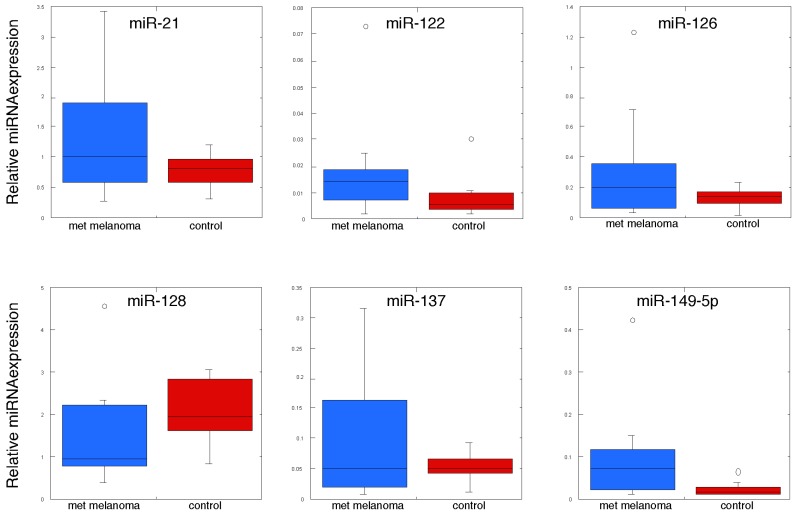
MiRNA expression in plasma-derived exosomes from individuals with metastatic melanoma and normal volunteers. RNA was prepared from plasma-derived exosomes from individuals with metastatic melanoma and normal volunteers. MiRNA expression was determined by qPCR (*n* = 3) and normalized to the spiked-in levels of Cel39.

We then examined the expression of circulating miRNAs by qPCR using the RNAs derived from the original cohort, plus an additional 3 metastatic melanoma patient samples. Thus, we analyzed miRNA expression in 13 individuals with metastatic melanoma, 13 control volunteers, 5 individuals with the p16 mutation (CDKN2A:c.377T>A (p.V126D)) but with no clinical evidence of melanoma incidence, and 8 individuals with the p16 mutation with melanoma. We subjected the qPCR data on the circulating miRNAs to statistical analysis as shown in Table 3, Table 4 and Table 5, and presented as “box plots” in Figure 2 and Figure 3. In Table 3 we compared the expression levels of these miRNAs between individuals with the CDKN2A:c.377T>A (p.V126D) mutation to those of normal volunteers, and found that there were no statistically significant differences between expression of any of the 20 exosomal miRNAs measured. In Table 4 and Figure 2 we show the comparison in miRNA expression between individuals with the p16 mutation (CDKN2A:c.377T>A (p.V126D)) that had no evidence of melanoma *versus* those individuals that had a history of melanoma. Most interestingly, expression of miR-125b was 1.5-fold higher in those individuals with the p16 mutation (CDKN2A:c.377T>A (p.V126D)) that had no evidence of melanoma as compared to individuals with this mutation that had a history of melanoma (*p* value of 0.025). In Table 5 and Figure 3 we show the comparison in miRNA expression between control individuals and patients with metastatic melanoma. Most interestingly, miR-17, miR-19a, miR-21, miR-126 and miR-149 were expressed at 1.8-fold, 2.3-fold, 1.7-fold, 2.8-fold and 3.9-fold higher levels, respectively, in patients with metastatic melanoma (*p* values of 0.044, 0.015, 0.038, 0.040 and 0.021, respectively).

**Table 3:** MiRNA expression in plasma-derived exosomes from p16 mutation carriers.

MiRNA	Control	p16 Carriers	*p* Value
hsa-let-7b	0.118 ± 0.001	0.104 ± 0.012	0.217
hsa-let-7g	0.056 ± 0.007	0.051 ± 0.011	0.350
hsa-miR-125b	1.319 ± 0.125	1.251 ± 0.150	0.368
hsa-miR-126	0.113 ± 0.019	0.127 ± 0.018	0.425
hsa-miR-128	2.034 ± 0.210	1.826 ± 0.206	0.242
hsa-miR-137	0.052 ± 0.005	0.045 ± 0.006	0.252
hsa-miR-148a	0.094 ± 0.011	0.083 ± 0.008	0.224
hsa-miR-149	0.024 ± 0.004	0.028 ± 0.006	0.310
hsa-miR-17	0.101 ± 0.17	0.097 ± 0.013	0.418
hsa-miR-199a	0.017 ± 0.004	0.016 ± 0.010	0.451
hsa-miR-19a	0.421 ± 0.067	0.409 ± 0.053	0.446
hsa-miR-19b	0.558 ± 0.090	0.543 ± 0.077	0.450
hsa-miR-20b	0.123 ± 0.020	0.107 ± 0.012	0.273
hsa-miR-21	0.775 ± 0.074	0.789 ± 0.054	0.441
hsa-miR-221	0.335 ± 0.030	0.311 ± 0.023	0.273
hsa-miR-222	0.589 ± 0.062	0.519 ± 0.062	0.182
hsa-miR-23a	0.520 ± 0.095	0.473 ± 0.072	0.350
hsa-miR-29a	0.625 ± 0.054	0.593 ± 0.044	0.323
hsa-miR-423-3p	0.088 ± 0.014	0.078 ± 0.006	0.264
hsa-miR-92a	0.341 ± 0.053	0.306 ± 0.034	0.292

**Table 4:** MiRNA expression in plasma-derived exosomes from p16 mutation carriers with or without melanoma.

MiRNA	p16 No Melanoma	p16 with Melanoma	*p* Value
hsa-let-7b	0.119 ± 0.017	0.094 ± 0.016	0.162
hsa-let-7g	0.048 ± 0.007	0.052 ± 0.018	0.422
hsa-miR-125b	1.571 ± 0.081	1.052 ± 0.214	0.025
hsa-miR-126	0.140 ± 0.007	0.120 ± 0.030	0.274
hsa-miR-128	1.908 ± 0.223	1.774 ± 0.315	0.368
hsa-miR-137	0.036 ± 0.004	0.051 ± 0.009	0.103
hsa-miR-148a	0.089 ± 0.004	0.079 ± 0.013	0.256
hsa-miR-149	0.021 ± 0.002	0.032 ± 0.010	0.172
hsa-miR-17	0.111 ± 0.012	0.088 ± 0.021	0.187
hsa-miR-199a	0.020 ± 0.006	0.013 ± 0.002	0.183
hsa-miR-19a	0.404 ± 0.037	0.411 ± 0.087	0.471
hsa-miR-19b	0.485 ± 0.053	0.579 ± 0.123	0.252
hsa-miR-20b	0.121 ± 0.016	0.099 ± 0.018	0.191
hsa-miR-21	0.757 ± 0.046	0.809 ± 0.085	0.301
hsa-miR-221	0.354 ± 0.029	0.285 ± 0.031	0.068
hsa-miR-222	0.577 ± 0.046	0.483 ± 0.061	0.125
hsa-miR-23a	0.542 ± 0.069	0.430 ± 0.110	0.205
hsa-miR-29a	0.544 ± 0.061	0.623 ± 0.061	0.193
hsa-miR-423-3p	0.094 ± 0.012	0.068 ± 0.005	0.063
hsa-miR-92a	0.382 ± 0.075	0.258 ± 0.018	0.088

**Table 5:** MiRNA expression in plasma-derived exosomes from patients with metastatic melanoma.

MiRNA	Control	Metastatic Melanoma	*p* Value
hsa-let-7b	0.118 ± 0.001	0.192 ± 0.066	0.146
hsa-let-7g	0.056 ± 0.007	0.065 ± 0.027	0.378
hsa-miR-125b	1.319 ± 0.125	1.219 ± 0.468	0.420
hsa-miR-126	0.113 ± 0.019	0.320 ± 0.096	0.040
hsa-miR-128	2.034 ± 0.210	1.420 ± 0.322	0.063
hsa-miR-137	0.052 ± 0.005	0.102 ± 0.030	0.067
hsa-miR-148a	0.094 ± 0.011	0.126 ± 0.028	0.150
hsa-miR-149	0.024 ± 0.004	0.094 ± 0.030	0.021
hsa-miR-17	0.101 ± 0.17	0.181 ± 0.040	0.044
hsa-miR-199a	0.017 ± 0.004	0.028 ± 0.006	0.084
hsa-miR-19a	0.421 ± 0.067	0.986 ± 0.222	0.015
hsa-miR-19b	0.558 ± 0.090	1.203 ± 0.290	0.259
hsa-miR-20b	0.123 ± 0.020	0.202 ± 0.046	0.071
hsa-miR-21	0.775 ± 0.074	1.305 ± 0.268	0.038
hsa-miR-221	0.335 ± 0.030	0.390 ± 0.085	0.279
hsa-miR-222	0.589 ± 0.062	0.680 ± 0.123	0.258
hsa-miR-23a	0.520 ± 0.095	0.773 ± 0.208	0.142
hsa-miR-29a	0.625 ± 0.054	0.795 ± 0.150	0.154
hsa-miR-423-3p	0.088 ± 0.014	0.082 ± 0.010	0.369
hsa-miR-92a	0.341 ± 0.053	0.267 ± 0.036	0.133

**Figure 4:**
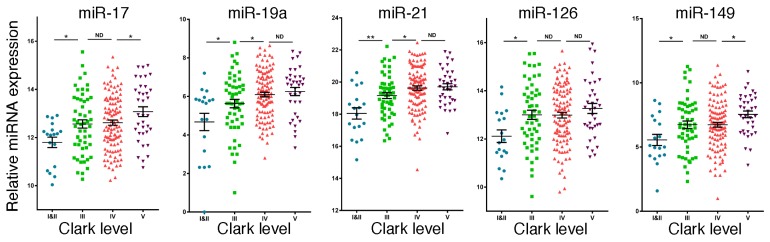
High expression of miR-17, miR-19a, miR-21, miR-126 and miR-149 is associated with melanoma tumor grade. Expression of miR-17, miR-19a, miR-21, miR-126 and miR-149 in the TCGA database for 216 independent melanoma patient samples according to Clark level (Level 1 is the least aggressive and Level V is the most aggressive).

### 2.3. Expression of Potential miRNA Biomarkers in Melanoma

Based on the results from the above studies, we then sought to determine whether miRNAs, which were differentially expressed in plasma exosomes derived from patients with metastatic melanoma, were also differentially expressed in melanoma tumor tissue. Therefore, we examined the TCGA database for miRNA expression in 216 melanoma specimens, which were classified according to Clark level (level I/II is minimally invasive cancer and level V is the most highly invasive form). As shown in Figure 4, low expression of miR-17, miR-19a, miR-21, miR-126 and miR-149 was found in thinner melanoma (Clark level I/II) and high expression was found in thicker melanoma (Clark level III, IV and V). These results provide additional evidence that these exosomal miRNAs are associated with the occurrence of melanoma *in situ*. Such melanoma samples include the tumor cells as well as the cells in tumor microenvironment that coordinately regulate tumorigenesis.

### 2.4. The Potential Biological Functions of the miRNAs Upregulated in Metastatic Melanoma

**Figure 5:**
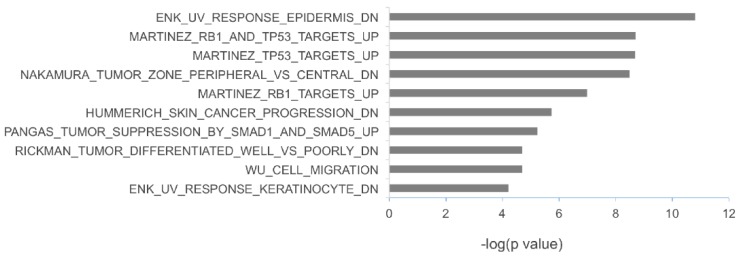
Enriched gene sets of putative targets of miRNAs upregulated in metastatic melanoma. The conserved target sites of miR-17-5p, miR-19a-3p, miR-149-5p, miR-21 and miR-126-3p were mapped to the 3-UTRs of a panel of genes that have been associated with melanoma progression according to TargetScan V6.2. Gene set enrichment analysis of putative miRNA targets was conducted by using the Molecular Signatures Database v5.0.

To investigate the potential biological functions of the miRNAs upregulated in metastatic melanoma, the target sites of miR-17-5p, miR-19a-3p, miR-149-5p, miR-21 and miR-126-3p were mapped to the 3-UTRs of a panel of genes that have been previously found to be associated with melanoma progression [21,22,23]. Forty genes associated with melanoma progression were found to be putative targets of these miRNAs (Table 6). To gain insights into the biological pathways that these putative miRNA targets may affect, we performed gene set enrichment analysis using the Molecular Signatures Database v5.0 [24], which is a computational method used to identify over-represented gene sets with defined biological meanings [25]. As shown in Figure 5, the most significantly enriched gene sets contain genes downregulated by ultraviolet (UV) irradiation (*i.e.*, genes included in the ENK_UV_RESPONSE_EPIDERMIS_DN and ENK_UV_RESPONSE_KERATINOCYTE_DN gene sets), targeted by the tumor protein p53 (TP53)/retinoblastoma protein (RB1) (*i.e.*, genes included in the MARTINEZ_RB1_AND_TP53_TARGETS_UP, MARTINEZ_RB1_TARGETS_UP and MARTINEZ_TP53_TARGETS_UP gene sets) and genes related to the tumor growth factor-beta (TGFB)/SMAD pathways (*i.e.*, genes included in the PANGAS_TUMOR_SUPPRESSION_BY_SMAD1_AND_SMAD5_UP gene set).

**Table 6:** Putative targets of miRNAs upregulated in metastatic melanoma.

Genes		miR-17	miR-19a	miR-149	miR-21
ADD3	adducin 3 (gamma)			y	
ARL4C	ADP-ribosylation factor-like 4C	y			
BCL11A	B-cell CLL/lymphoma 11A (zinc finger protein)		y		y
BCL11B	B-cell CLL/lymphoma 11B (zinc finger protein)	y			y
CD34	CD34 molecule			y	
CDS1	CDP-diacylglycerol synthase (phosphatidate cytidylyltransferase) 1		y		
CXCL12	chemokine (C-X-C motif) ligand 12 (stromal cell-derived factor 1)		y		
CYBB	cytochrome b-245, beta polypeptide	y			
DSC3	desmocollin 3			y	
EREG	epiregulin	y	y		
ESR1	estrogen receptor 1	y	y		
FAT2	FAT tumor suppressor homolog 2	y			
FBLN1	fibulin 1	y		y	
GJA1	gap junction protein, alpha 1, 43 kDa	y	y		
GRHL2	grainyhead-like 2 (Drosophila)	y			
HLF	hepatic leukemia factor	y	y		
ID2	inhibitor of DNA binding 2, dominant negative helix-loop-helix protein		y		
LRIG1	leucine-rich repeats and immunoglobulin-like domains 1	y	y		
LRRK1	leucine-rich repeat kinase 1		y		
LTB4R	leukotriene B4 receptor			y	
MACF1	microtubule-actin crosslinking factor 1		y		
MBNL1	muscleblind-like (Drosophila)	y	y		y
MGEA5	meningioma expressed antigen 5 (hyaluronidase)	y			
MPZL2	myelin protein zero-like 2	y			
NFATC3	nuclear factor of activated T-cells, cytoplasmic, calcineurin-dependent 3			y	
NLRP3	NLR family, pyrin domain containing 3	y			
NMT2	N-myristoyltransferase 2		y		
NTRK2	neurotrophic tyrosine kinase, receptor, type 2	y		y	
PAIP2B	poly(A) binding protein interacting protein 2B			y	y
PTGER3	prostaglandin E receptor 3 (subtype EP3)	y			
PTGS1	prostaglandin-endoperoxide synthase 1 (prostaglandin G/H synthase and cyclooxygenase)	y			
RAPGEFL1	Rap guanine nucleotide exchange factor (GEF)-like 1	y	y		
RORA	RAR-related orphan receptor A	y	y	y	
RTN1	reticulon 1		y		
TCF4	transcription factor 4	y			
TMEM45A	transmembrane protein 45A		y		
TNFRSF25	tumor necrosis factor receptor superfamily, member 25			y	
TP63	tumor protein p63	y		y	
TXNIP	thioredoxin interacting protein	y			
ZFP36L2	zinc finger protein 36, C3H type-like 2				y

No binding sites of miR-126 were found in the 3′-UTRs of the genes associated with melanoma progression.

## 3. Discussion

To date, over 1800 human miRNAs have been identified, and miRNAs are predicted to control over 60% of all human genes [2,26]. MiRNAs regulate a wide variety of cellular processes, including cancer. The miRNAs differentially expressed in patients with metastatic melanoma may play important roles in tumor progression and metastasis, as well as be explored as diagnostic biomarkers. Exosomal miRNAs may transfer or shuttle signals between cancer cells and normal cells, and may contribute to malignant transformation. Differentially-expressed exosomal miRNA has now been demonstrated for many forms of cancer [27], and a recent study has shown that a panel of 5 miRNAs can be used to estimate risk of recurrence in stage II melanoma patients [28]. The measurement of tumor-derived miRNAs in serum or plasma may be an important approach to cancer detection [20]. In a previous study, higher levels of circulating miR-221 were found in serum samples of malignant melanoma patients as compared to healthy volunteers [29].

In the present study, we investigated miRNA signatures of plasma-derived exosomes from familial and sporadic melanoma patients and unaffected family members. Several miRNAs were differentially expressed in plasma-derived exosomes, which may form the basis for future studies on their applicability as predisposition biomarkers and potential chemoprevention targets. An important aspect of our studies was the finding that miR-17, miR-19a, miR-21, miR-126 and miR-149 were expressed at higher levels in plasma-derived exosomes from patients with metastatic melanoma. Many of these miRNAs have been associated with various cancers, and in some cases with melanoma specifically. For example, using a high-throughput approach, miR-17 was identified as a potential oncogenic miRNA in melanoma [30]. Previous studies demonstrated that miR-17 is highly expressed in leukemia and lung cancer, and it promotes cell proliferation by targeting p21 [31,32] as well as PTEN and RB [33,34]. Also, increased expression of miR-19a leads to increased melanoma invasiveness [35]. MiR-19a is an important member of the oncogenic miR-17-92 cluster. MiR-19a is upregulated in acute myeloid leukemia, colorectal cancer and gastric cancer, and is believed to act through promoting tumor growth and metastasis [36,37]. MiR-21 is frequently upregulated in human tumor cells where it appears to play an important role in the oncogenic process through its association with increased proliferation, low apoptosis, high invasion and metastatic potential [38,39,40,41,42,43,44]. However, miR-21 is also upregulated in the inflammatory response, which also may play an important role in tumor progression as well as in tumor elimination (reviewed in [45]). We recently found that IFN upregulated miR-21 expression in both melanoma and prostate cancer cells, which diminished their apoptotic sensitivity [46,47]. In contrast, knockdown (KD) of miR-21 expression enhanced apoptotic sensitivity to IFN as well as to several chemotherapeutic agents. Consistent with these findings, miR-21 inhibition in human melanoma cells increases expression of the PTEN target gene, leading to suppression of AKT phosphorylation and subsequently increased Bax/Bcl-2 ratio [48]. Most interestingly, using mouse B16 melanoma, we found that while the parent cell line exclusively formed large tumors in the lungs of tail-vein injected mice, miR-21 KD cells formed only small lung tumors [47], and mice injected with miR-21 KD cells exhibited markedly prolonged animal survival. Elevated miR-126 expression has been observed in normal melanocytes and primary melanoma cell lines, while it was reportedly reduced in metastatic melanoma [49]. Overexpression of miR-126 was found to enhance melanogenesis. MiR-149 is upregulated in melanoma cells and is expressed in response to p53 activation [50]. However, miR-149 provides a mechanism to bypass the induction of apoptosis by p53 activation by directly targeting glycogen synthetase-3α and thereby stabilizing MCL-1.

Consistent with the reported function of these miRNAs in regulating cell proliferation and metastatic potential, target site mapping to genes associated with melanoma progression suggests that miR-17-5p, miR-19a-3p, miR-149-5p and miR-21 play a role in modulating cell response to TP53/RB1 activation and TGFβ/SMAD signaling pathways. Since these pathways play an important role in regulating the G1/S checkpoint of normal melanocytes and are major regulators of melanocyte transformation [51,52], the upregulation of miRNAs controlling TP53/RB1 activation and TGFβ/SMAD signaling pathways may contribute to G1/S checkpoint abnormalities that are frequently observed during melanoma progression. Since these signaling pathways are critical to the malignant process, further studies will be needed to define the roles of these pathways directly in melanoma cells themselves and in the stromal cells surrounding the tumor.

Although we hypothesized that genetically predisposed familial melanoma patients with/without evidence of disease might share their miRNA expression profile with sporadic metastatic melanoma patients, no major differences between p16 mutation (CDKN2A:c.377T>A (p.V126D)) gene carriers and normal controls were detected. There are several possible explanations for our inability to discern miRNAs that are risk biomarkers in cohorts of familial melanoma. These possibilities include: (1) differences in miRNA profiles of the cohorts in our p16 families are too small to be detected with the sample size available in this work (although relative to the uniqueness of the cohort, the number of samples used in this study is large); (2) the miRNA profile of an affected p16 mutation carrier (CDKN2A:c.377T>A (p.V126D)) is not significantly different from a carrier that has not had a melanoma or from a normal control; (3) other p16 mutations such as the CDKN2A:c.259G>T (p.R87P) mutation [16,53] may more closely share the miRNA pattern associated with the pattern observed in plasma-derived exosomes from sporadic malignant melanoma patients; and (4) the metastatic patients may have had circulating tumor cells which contributed and further amplified the differences seen in exosomes, and tumor exosomes are different from those that might be associated with genetic predisposition to melanoma. Our findings are consistent with the occurrence of miRNA dysregulation in metastatic melanoma. Therefore, this technology might be better suited for detecting recurrent metastatic melanoma following therapeutic intervention.

Taken together, our results show that we have been able to identify several circulating miRNAs that are up-regulated in plasma-derived exosomes from patients with sporadic metastatic melanoma. While the increased expression of these unique miRNAs in the plasma-derived exosomes may be due, in part, to the occurrence of tumor cells in the circulation, these circulating miRNAs have prognostic potential in patients with metastatic melanoma. Future studies should be directed at discerning the role of these individual miRNAs in melanoma progression and metastasis, particularly in response to therapy.

## 4. Materials and Methods

### 4.1. Plasma

Archived citrate-treated plasma samples from 8 individuals with CDKN2A/p16 mutations with a history of melanoma, 5 individuals with CDKN2A/p16 mutations with no history of melanoma, 13 spouse controls and 10 patients with metastatic melanoma were obtained from the Huntsman Cancer Institute of the University of Utah. All subjects gave their informed consent for inclusion before they participated in the study. The study was conducted in accordance with the Declaration of Helsinki, and the protocol was approved by the Ethics Committee of the University of Utah (7916-00). General clinical patient information is shown in Table 1.

### 4.2. Preparation of Plasma-Derived Exosomes and Isolation of RNA

Following a protocol previously established in our group [8], citrate-treated plasma was incubated at 37 °C for 15 min with Thromboplastin-D to remove clotting factors, and centrifuged (8000× *g* for 15 min at 22 °C). The resultant supernatant (1.2 mL) was mixed by inversion with 140 μL of ExoQuick solution (System Biosciences, Mountain View, CA, USA) overnight at 4 °C. The ExoQuick/plasma mixture was centrifuged (1500× *g* for 15 min at 22 °C), and the exosomal pellet was washed twice with PBS. The exosomal pellet was resuspended in 700 µL of Qiazol (containing 0.05 fmol/mL of Cel39 for miRNA normalization) and incubated for 5 min at 22 °C. The RNA was extracted by addition of 140 μL of chloroform by incubating for 5 min at RT. After centrifugation at 12,000× *g* for 15 min at 4 °C, 280 μL of the upper aqueous phase was transferred to a new tube, mixed with 420 μL of 100% ethanol and loaded on a miRNeasy MinElute spin column. After centrifugation at 8000× *g* for 15 s at 22 °C, the column was washed three times with RNeasy buffer RWT followed by centrifugation. The RNA was concentrated to a final volume of 20 μL with RNase-free water with an Amicon Ultra YM-3 filter by centrifugation at 14,000× *g* at 22 °C for 45 min.

### 4.3. miRNA Expression Profiling

Microarray analysis on RNA prepared from plasma-derived exosomes was performed using the human V1 miRNA assay kit (NanoString Technologies, Seattle, WA, USA) that contains ~700 human miRNAs. The integrity and quantity of the RNA was assessed using the Agilent 2100 Bioanalyzer and RNA 6000 Pico Kit (Agilent Technologies, Santa Clara, CA, USA). In brief, total RNA was mixed with pairs of capture and reporter probes, hybridized on the nCounter Prep Station, and purified complexes were quantified on the nCounter digital analyzer. To account for differences in hybridization and purification, data were normalized to the average counts for all control spikes in each sample and analyzed with nSolver software.

### 4.4. miRNA Expression Using SYBR Green Real Time PCR

PolyA-tailed total RNA was prepared from plasma-derived exosomes using (polyA) polymerase (NEB; Ipswich, MA, USA) at 37 °C for 1 h as previously described [46]. The final reaction mixture was extracted with phenol/chloroform, precipitated with isopropanol, redissolved in diethylpyrocarbonate (DEPC)-treated water, and was reverse-transcribed into first-strand cDNA using Superscript III transcriptase (Invitrogen) with the oligo-dT adapter primer 5′GCGAGCACAGAATTAATACGACTCACTATAGGTTTTTTTTTTTTVN3′. For PCR, 40 ng of cDNA was used as a template in each reaction. The reverse primer was from the adapter sequence: 5′GCGAGCACAGAATTAATACGACTCAC3′ and the forward primers were specific to miRNA mature sequences (shown in Table 2). The SYBR Green-based real-time PCR was performed to quantify miRNA expression, and Cel39 was used to normalize miRNA expression. The expression data was normalized to the expression of the spiked in C. elegans miRNA (Cel39) as a normalizing control as previously described [20].

### 4.5. TCGA Data Query

To examine the relationship between miR-17, miR-19a, miR-21, miR-126 and miR-149 expression in human cancer specimens from cutaneous melanoma, we queried the TCGA data portal [54] for all samples with Level 3 miRNA expression data available, as well as the accompanying clinical data. The data set was filtered for samples having expression data for these selected miRNAs and clinical data, yielding a final set of 216 melanoma independent patient samples.

### 4.6. Statistical Analysis

At least two independent PCR experiments were performed in triplicate, and data are presented as means ± sd. ANOVA and post-hoc least significant difference analysis or Student *t* tests were performed using Graphpad InStat 3 software, with *p*-values < 0.05 considered statistically significant.
